# Slightly and Moderately Lame Cows in Tie Stalls Behave Differently From Non-lame Controls. A Matched Case-Control Study

**DOI:** 10.3389/fvets.2020.594825

**Published:** 2020-12-17

**Authors:** Johanna Karin Bernhard, Beatriz Vidondo, Rahel Lisa Achermann, Rahel Rediger, Dimitri Stucki, Kerstin Elisabeth Müller, Adrian Steiner

**Affiliations:** ^1^Clinic for Ruminants, Vetsuisse-Faculty, University of Bern, Bern, Switzerland; ^2^Veterinary Public Health Institute, Vetsuisse-Faculty, University of Bern, Bern, Switzerland; ^3^Clinic for Ruminants and Swine, Faculty of Veterinary Medicine, Freie Universität Berlin, Berlin, Germany

**Keywords:** lameness, behavior, tie stall, dairy cow, well-being, RumiWatch

## Abstract

Lameness affects dairy cows worldwide and is usually associated with pain. Behavioral differences in lame compared to non-lame tie-stall-housed dairy cows might be less pronounced than in free-stall-housed, since the principle demands to a cow's locomotor system and thus the impact of lameness on behavior seem to be lower in tie stalls. Behavioral differences between lame and non-lame cows might be used to estimate the impact of lameness on the well-being of tied dairy cows. In the current study, lame cows were categorized as locomotion scoring between 2.25 and 3.25 on a 1–5 scale. The aim was to compare the eating, rumination and lying behavior of lame cows against non-lame tied dairy cows, in order to draw conclusions on the association of lameness, behavior and well-being in tied dairy cows. The eating and rumination behavior of 26, the lying behavior of 30, and the relative upright and lying activities of 25 matched case-control pairs were analyzed, considering the matching criteria farm, breed-type, and parity-group. Lame cows had fewer [mean of the pairwise differences (case–control) (mean_diff_) = −2.6 bouts, CI_95%_ (−3.8–−1.4) bouts, *p* = 0.001], but longer lying bouts [mean_diff_ = 26.7 min per bout, CI_95%_ (10.1–43.4) min per bout, *p* = 0.006]. The lying time was shorter [mean_diff_ = −64.7 min, CI_95%_ (−104.4–−24.9) min, *p* = 0.006] in lame cows compared to their non-lame controls. Lame cows had a shorter eating time [mean_diff_ = −27.7 min, CI_95%_ (−51.5–−4.0) min, *p* = 0.042] and spent a larger proportion of their upright time ruminating [mean_diff_ = 7.2%, CI_95%_ (3.2–11.1)%, *p* = 0.001] instead of eating. The results of the current study indicate that the eating, rumination, and lying behavior of lame tied dairy cows is altered. These findings indicate that slight and moderate lameness (locomotion score between 2.25 and 3.25 on a 1–5 scale) are likely to be associated with an impaired well-being in affected tied dairy cows. This underlines the need to continuously reduce the lameness prevalence and severity in tied dairy herds.

## Introduction

Lameness affects free-stall-housed and tied dairy cows worldwide. It usually is a painful condition (1, 2) caused by a variety of orthopedic diseases and resulting in cows changing their gait. Lameness can lead to considerable changes of eating, rumination, and locomotion behavior (3–5). The reported high prevalence of lameness within the past decade, ranging from 8 to 55% in free (6–9) and 15 to 25% in tie stall herds (9–12) and the potentially long disease duration make lameness one of the most important welfare issues in dairy cows (13).

The demands to a dairy cow's locomotor system largely differ between housing systems. Free-stall-housed dairy cows need to walk on an artificial floor, such as slatted concrete or rubber mats to reach the feeder, drinking trough and milking parlor; additionally, long waiting periods on inflexible, solid surfaces might occur prior to milking. Tie-stall-housed dairy cows stand in their individual stalls and obtain their individual feeding place, water bowl, and lying area; thus, they do not need to cover long distances walking to fulfill their daily needs. The demands to tied cows' locomotor systems are mostly related to space restrictions, the inability of the cows to move freely and the tethering itself. For instance, “a very short tether may limit the cows' ability to lunge forward” (14), impeding rising and lying down. Although, lameness affects both tie and free-stall-housed cows, its influence on behavior and welfare might differ between the two housing systems.

Tie stalls are a topic of controversial discussions (15) and their construction and use is subordinated to a strict animal welfare ordinance: the permanent year-round tie-stall-housing of dairy cows is prohibited in Switzerland (16); additionally, most Swiss dairy farmers (73.6%) participate in a national program for regular outdoor exercise (17) and provide their herds at least 13 days of outdoor exercise per winter month and at least 26 days per summer month (18). Regular outdoor exercise is associated with a lower lameness prevalence (10, 19), might compensate some detriments of the tie stall housing system and increases the acceptance of tie stalls in public (20). At last count, 46% of Swiss stall places for cattle were tie stalls (21), and since traditionally managed tie stall herds play an important role in the agricultural branch of Alpine pasturing, it can be expected that a relevant proportion of Swiss dairy cows will further on be managed in tie stall systems within the next years.

Behavioral differences between sick and healthy cows are often used to estimate the impact of a certain disease or pathological state on well-being (14, 22) or to establish smart-farming devices for the detection of disease, such as lameness (23, 24). Automated lameness detection should focus on an early disease detection. A farmer could easily identify severely lame cows with an observation (25) but slightly lame cows are often detected and treated with a delay (26–28). In tie stalls, where the time to observe the cows' gait is limited to the periods of outdoor exercise, the correct and timely identification of lameness is suspected to be even more challenging compared to free stalls. The chances of full recovery are improved when slightly and moderately compared to severely lame cows are adequately treated (27, 28). Consequently, the time of a potential negative impact of the disease on the well-being of the cow could be shortened, when slightly lame cows are detected and treated and less likely to become chronically affected. It seems obvious that severe lameness affects a cow's well-being. Describing behavioral differences between slightly and moderately lame to non-lame tied dairy cows might provide further insight in the association between rather mild lameness and the well-being of tied dairy cows. Additionally, behavioral differences might serve as a basis for automated lameness detection for tied dairy cows in future. In free stalls, lame compared to non-lame cows spend less time eating (5, 24, 29), but have a higher mastication speed (5, 24). Further, they spend less time ruminating (24, 29), and more time lying down (30, 31). In tie-stall housed dairy cows, the impact of lameness on behavior seems to be less pronounced. A recent study found no significant association between lameness and lying behavior at the cow level and related this to a large variation in lying behavior among individuals and farms (11). Indeed, diverse factors influence a cow's behavior, increase interindividual variation and need to be considered when making assumptions about the distinct impact of lameness on behavior and well-being. For instance, management and housing, breed (32), parity (32, 33), lactation stage (34, 35), and productivity (36) influence behavior and need to be accounted for. By comparing the behavior of lame and non-lame tied dairy cows from the same farm, breed-type [dairy vs. dual-purpose, (37)], and parity-group [(1) primiparous; (2) second to fourth lactation with a maximum deviation of ±1 within pairs; (3) ≥fifth lactation] in a matched case-control approach, some of the main influencing factors within a matched pair can be equalized. Thus, it gets more likely to correctly identify behavioral alterations arising from the lameness itself. We expected lame cows to have longer daily lying times and a lower lying bout frequency compared to their non-lame controls, in order to disburden the painful limb and to avoid the potentially painful procedure of rising and lying down. We expected the eating and ruminating time of lame cows to be shorter compared to their non-lame controls, as lame cows suffer from pain and might, therefore, have a reduced appetite.

Our objective was to create a deeper understanding of the impact of slight and moderate lameness on the behavior of tied dairy cows, in order to draw conclusions on affected cows' well-being. Therefore, lame cows were categorized as locomotion scoring between 2.25 and 3.25 on a 1 to 5 scale. The aim of the current study was to apply a matched case-control design to compare the eating, rumination and lying behavior and the jaw activities (idling, eating, ruminating) performed in either upright or lying posture of lame to non-lame tied dairy cows.

## Materials and Methods

All farmers participated voluntarily after being informed via newspaper articles and information letters, which were sent to the members of the Swiss tie stall association. The primary inclusion criterion for farms was the housing of at least 15 lactating dairy cows in a tie stall. Data were collected between December 2017 and April 2018, and between November 2018 and March 2019. All farms were visited twice within 4 to 5 days. The mean herd size of the enrolled herds was 29, with a median (interquartile range) of 29 (20–38) and a range of 16–55 cows per herd. All lactating cows were milked twice daily in-stall. The average milk yield of the cows could not be assessed, as not all farmers had the technical possibilities to record individual milk yield or participated in an official milk-recording program. Apart from the study period, the cows had a median (interquartile range) of 13 (13–14) days of outdoor access per month. Cows under current medication or under the withdrawal period of any type of medication were excluded from the study.

The overall objective of the cow-selection procedure comprising on and off-farm procedures was to build matched case-control pairs, each formed by one lame and one non-lame cow of the same farm, breed-category, and parity-group. A flowchart of the cow-selection procedure is given in Supplementary Figure 1.

### On-Farm Assessment

To gain an overview of the cows' lameness states and to preselect potentially eligible cows for more elaborate scoring and examination methods, cows were examined for their live stall locomotion score (SLS) (38). Each cow was individually observed in its stall for the presence of the defined behaviors weight shifting, standing at the edge of the rear curb, resting a foot while standing, and unequal weight bearing while stepping from side to side. If at least two of these behaviors were recorded within the observation period of 90 s, the cow was considered lame according to the SLS (*n* = 88), and if not, she was considered non-lame according to the SLS (*n* = 589). The SLS was not included in the statistical analyses but was used as a preselection criterion. Potentially eligible cows to be included in the matching process were selected from the herd depending on their breed-category, parity-group and SLS, and walked by the farmer in a straight line on a solid, non-slippery floor. The gait was videotaped laterally; simultaneously, the first author applied a live locomotion scoring procedure (live LS) as described by Flower and Weary (39). This scoring system considers the gait characteristics back arch, head bob, tracking-up, joint stiffness, asymmetry of gait, and equal weight bearing, so that each cow's locomotion can be classified on a 5-point-scale with half-point intervals (1.0 = sound; 5 = severely restricted ability to move). For instance, a score of 2.5 can be allocated to those cows that exceed the requirements of score 2.0 (cow with an imperfect gait but an unaffected ability to move freely), but do not meet all the criteria of score 3.0 (capable of locomotion but ability to move freely is compromised). Cows with a live LS of ≥ 2.5 were considered as potential cases; cows with a live LS of < 2.5 as potential controls (24). Severely lame cows (live LS ≥ 4, *n* = 1) were excluded from the study, and the immediate consultation of a veterinarian was suggested.

Cows confirmed eligible due to their live LS were clinically examined (40) on both days of visit to ensure that their behavior was not influenced by a disease other than lameness. Any sign of disease, including a rectal temperature ≥ 39.0°C, cardiac murmurs, clinically relevant infection of the respiratory tract, gastrointestinal or nervous disorders, purulent vaginal discharge, or a painful, swollen udder led to the exclusion of the cow from the study (*n* = 2). Each cow's body condition score was recorded (41) and an EDTA-whole blood sample of eligible cows ≤ 90 days post-partum (*n* = 47) was analyzed for its content of beta-hydroxybutyrate using an electronic cowside test (Free Style Precision Neo, Abbot Diabetes Care Ltd., Oxfordshire, United Kingdom). Cows with subclinical ketosis (≥ 1.2 mmol/l beta-hydroxybutyrate, *n* = 12) were excluded from the study (42).

A total of 189 cows was included in the study and equipped with a RumiWatch® halter, incorporating a noseband sensor and an accelerometer, and a RumiWatch® pedometer, incorporating an accelerometer. The pedometer was attached to the left or right metatarsus; the side was selected haphazardly. After an adaption period of ≥12 h, each cow's eating, rumination, and lying behavior was recorded for 48 consecutive hours. During the period of data collection, farmers were obliged to record any sign of heat (*n* = 0) or disease (*n* = 0); which would have led to the exclusion of the respective cow from the study. The whole herd was kept in the tie stall with no outdoor exercise during the period of data collection.

### Off-Farm Assessment

To exclude a potential observer bias of the first author during the live LS procedure, all live LS were dismissed and replaced by video-based locomotion scores (39) of two independent, experienced researchers who were blinded to the cows' SLS and live LS. A difference of ≤ 0.5 between the two individual observers was accepted. If the deviation between the two observers was > 0.5 (*n* = 29), both observers independently scored the videos again; if the deviation remained > 0.5 (*n* = 4), the two observers met and found consensus by scoring the respective videos together. The scores of the two observers were averaged for the final locomotion score (final LS), which was considered in the matching process and all further analyses. Cows with a final LS < 2.25 were considered non-lame (*n* = 149 cows), cows with a final LS of 2.25 to 3.25 were considered lame (*n* = 37). Due to the low number of slightly (final LS of 2.25 to 2.75; *n* = 24) and moderately (final LS of 3.0–3.25, *n* = 13) lame cows identified, it was not possible to consider these separately in the analysis. Thus, both slightly and moderately lame cows were considered as “lame” in the analysis.

Finally, case-control pairs, formed by one non-lame control and one lame case, were matched. Both cows of a matched pair needed to belong to the same farm, breed-category and parity-group. Breeds were grouped into dairy breeds (Holstein-Friesian, Red Holstein, Brown Swiss) and dual-purpose breeds (Original Brown, Simmental, Swiss Fleckvieh) (37). The parity-groups were defined as follows: (1) primiparous; (2) second to fourth lactation with a maximum deviation of ±1 within pairs; (3) ≥ fifth lactation. Based on previously published literature, additionally matching the pairs on the criteria days post-partum, BCS, and milk yield would have been desirable. However, due to the typically small herd size in Swiss tie stalls, a low lameness prevalence, and a lack of technical equipment to measure individual milk yield in some herds, these factors were not included in the matching process. Days post-partum and BCS were recorded and considered in the statistical analyses.

### Data Handling

The raw data from the halters and pedometers were transferred to a personal computer and converted with specialized software (RumiWatch Manager 2, V2.2.0.0., and RumiWatch Converter, V0.7.3.6, ITIN + Hoch GmbH Fütterungstechnik, Liestal, Switzerland). The RumiWatch® pedometer differentiates lying, standing, and walking behavior (43); however, the walking behavior of tied dairy cows only consists of a few steps back and forth, thus, is very short [median walking time (interquartile range) of enrolled cows = 0.92 (0.56–1.67) min per day]. Therefore, standing and walking time were summed up to the variable upright time. The RumiWatch® halter differentiates eating, rumination, drinking, and other jaw movements (44). Since the correct detection of drinking behavior was not validated for the water bowls present in tie stalls, the time spent for drinking time and other jaw movements was summed up to the variable idle time. Definitions of the assessed behavioral parameters are given in Supplementary Table 1.

From the consecutive 48 h of raw data, 24 h summaries were created. The values of the following variables were averaged to create one average 24 h value: idle time, eating time, number of eating chews, rumination time, number of chews per rumination bolus, lying time, and lying bout frequency. Additionally, the mean lying bout duration (lying time divided by lying bout frequency), and the mean mastication speed (number of eating chews divided by eating time) were calculated.

To investigate if the time spent for each type of jaw activity (idling, eating, ruminating) performed in either upright or lying posture is associated with lameness, the jaw activities idling, eating, and ruminating were allocated to the posture (upright or lying) recorded at the same date and time at the level of 5 min intervals. All 5 min intervals during which a change from lying to upright or vice versa occurred, were omitted from the calculation, as the specific allocation of a jaw activity type to an either upright or lying position could not be guaranteed for these. The sum of the upright and lying time was depending on the individual lying bout frequency, hence, absolute time measures were not appropriate for a comparison between cows. Consequently, the proportions of time spent performing each jaw activity among the time spent in either upright or lying posture (Idle_up_, Eat_up_, Rumi_up_, and Idle_down_, Eat_down_, Rumi_down_) were calculated on the cow level and included in the analysis.

### Statistical Analysis

The statistical analysis was conducted in R (Version 3.6.3, 2020 The R Foundation for Statistical Computing) using the packages base (45), dplyr (46), ggplot2 (47), ggpubr (48), PairedData (49), and psych (50). Results with a *p*-value equal to or below a level of significance α = 0.05 were considered significant.

Since the case-control pairs were not matched for age, lactation stage, and BCS, paired Wilcoxon Rank Sum tests were used to investigate, if there were significant differences regarding these clinical parameters within pairs between cases and controls. The mean of the pairwise differences (case–control) (mean_diff_) and the respective confidence intervals were used to evaluate the clinical relevance of the statistical results. The confidence intervals were estimated using a bootstrap procedure with 500 repetitions.

The behavioral outcomes were reviewed for plausibility, investigated using descriptive statistics, and visually evaluated for their quality. Paired boxplots were used to visually investigate the data and potential associations between the behavioral outcomes and lameness within pairs. Because the behavioral outcomes may show some collinearity, pretests were conducted to inspect the validity of the data for a principle component analyses (PCA). Although, the determinant of the correlation matrix (determinant > 0.00001) and a significant Bartlett test (*p*-value < 0.001) indicated some correlation among the behavioral parameters, the Kaiser-Meyer-Olkin Criterion (KMO) and the Measure of Sampling Adequacy (MSA) dissuaded from performing a PCA (overall KMO = 0.38, range MSA = 0.22–0.56). Consequently, all behavioral outcomes were analyzed separately in paired *t*-tests to investigate differences between non-lame controls and lame cases. To account for testing multiple dependent variables regarding their dependence on lameness, the *p*-values resulting from the separate *t*-tests were adjusted using false discovery rates according to Benjamini and Hochberg (51).

To evaluate the effects of lameness (lame, non-lame), activity type (idling, eating, or ruminating), and posture (upright, lying) on the proportions of time spent performing each jaw activity, two separate repeated measure analyses of variance (rep. meas. ANOVA) were conducted (one for upright, one for lying posture). The interaction between activity type and posture was also included in the model. As a rep. meas. ANOVA only indicates significance for an entire factor, and not for each of its levels, unless there are only two, separate *post-hoc* paired *t*-tests were performed in case of a significant interaction term. As these tests were only applied to a subset of all possible pairwise comparisons, the resulting *p*-values were not adjusted for multiple comparisons (52).

## Results

### Study Population

**Figure 1:**
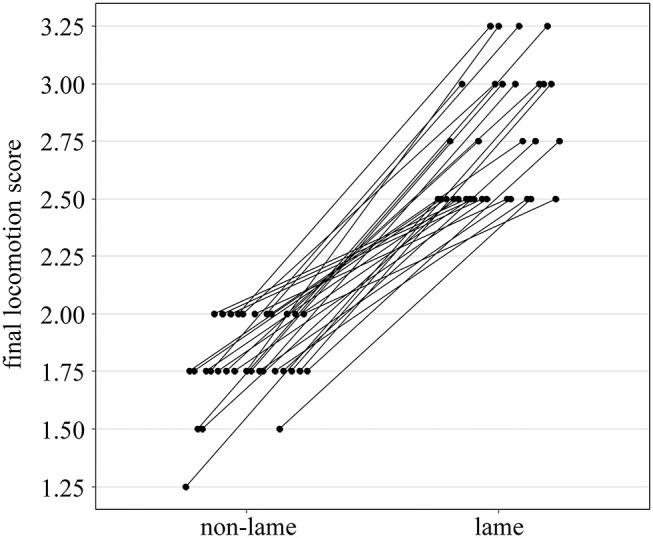
Final locomotion score of 31 non-lame (final locomotion score < 2.25) and 31 lame cows [final locomotion score ≥ 2.25, according to Flower and Weary (39)] included in the analyses. Each line between non-lame and lame represents a matched case-control pair.

Six of 37 lame cows were omitted from the analyses for lack of a non-lame control fulfilling the matching criteria. The analyzed dataset consisted of 31 matched pairs from 16 farms; each pair formed by one non-lame (control) and one lame cow (case). Due to missing data of four halters and one pedometer, and implausible data of one halter, 26 matched pairs from 15 farms were included in the analysis of idling, eating, and rumination, and 30 matched pairs from 16 farms in the analysis of lying behavior. For the analysis of Idle_up_, Eat_up_, Rumi_up_, and Idle_down_, Eat_down_, Rumi_down_, 25 matched pairs from 15 farms with complete halter and pedometer data were included. The distribution of final locomotion scores among matched pairs is presented in Figure 1.

### Clinical Variables and Behavioral Outcomes

The clinical variables are displayed in Table 1. The body condition score (41) of the lame cows was significantly lower compared to their non-lame controls (mean_diff_ = −0.13 score points, CI_95%_ [−0.21–−0.05] score points; paired Wilcoxon Rank Sum test: V = 26, *p*-value = 0.008). The lame cows had a tendentially lower number of days post-partum compared to their non-lame controls (mean_diff_ = −43.65 days, CI_95%_ [−95.49–11.94] days; paired Wilcoxon Rank Sum test: V = 151, *p*-value = 0.057).

**Table 1:** Clinical variables of non-lame (control) and lame (case) cows.

	**Non-lame (control)**^**a**^ **(*****n*** **=** **31)**						**Lame (case)**^**b**^ **(*****n*** **=** **31)**						
**Clinical variable^c^**	**Mean**	**SD^d^**	**Median**	**Min^e^**	**Max^f^**	**IQR^g^**	**Mean**	**SD^d^**	**Median**	**Min^e^**	**Max^f^**	**IQR^g^**	***P*-value^h^**
Final locomotion score	1.80	0.19	1.75	1.25	2.00	0.25	2.75	0.28	2.75	2.50	3.25	0.50	-
Parity	4.87	2.05	5.00	1.00	10.00	2.50	5.06	2.45	5.00	1.00	14.00	2.00	-
Age in years	7.12	2.20	7.06	2.44	12.25	2.70	7.33	2.54	7.41	3.15	16.28	2.41	0.710^h^
Days post-partum	208.68	97.86	206.00	45.00	424.00	127.50	165.03	115.33	182.00	5.00	496.00	143.50	0.057^h^
BCS	3.05	0.24	3.00	2.50	3.50	0.25	2.92	0.24	3.00	2.25	3.50	0.25	0.008^h^

a *Non-lame (control) = cows with a final locomotion score < 2.25, according to Flower and Weary (39)*.

b *Lame (case) = cows with a final locomotion score ≥ 2.25, according to Flower and Weary (39)*.

c *Clinical variable: Final locomotion score = average of video-based locomotion scores assessed by two independent observers, according to Flower and Weary (39); BCS = body condition score, according to Edmonson et al. (41)*.

d *SD = Standard deviation*.

e *Min = minimum*.

f *Max = maximum*.

g *IQR = Interquartile range*.

h *P-value of a paired Wilcoxon Rank Sum test*.

**Figure 2:**
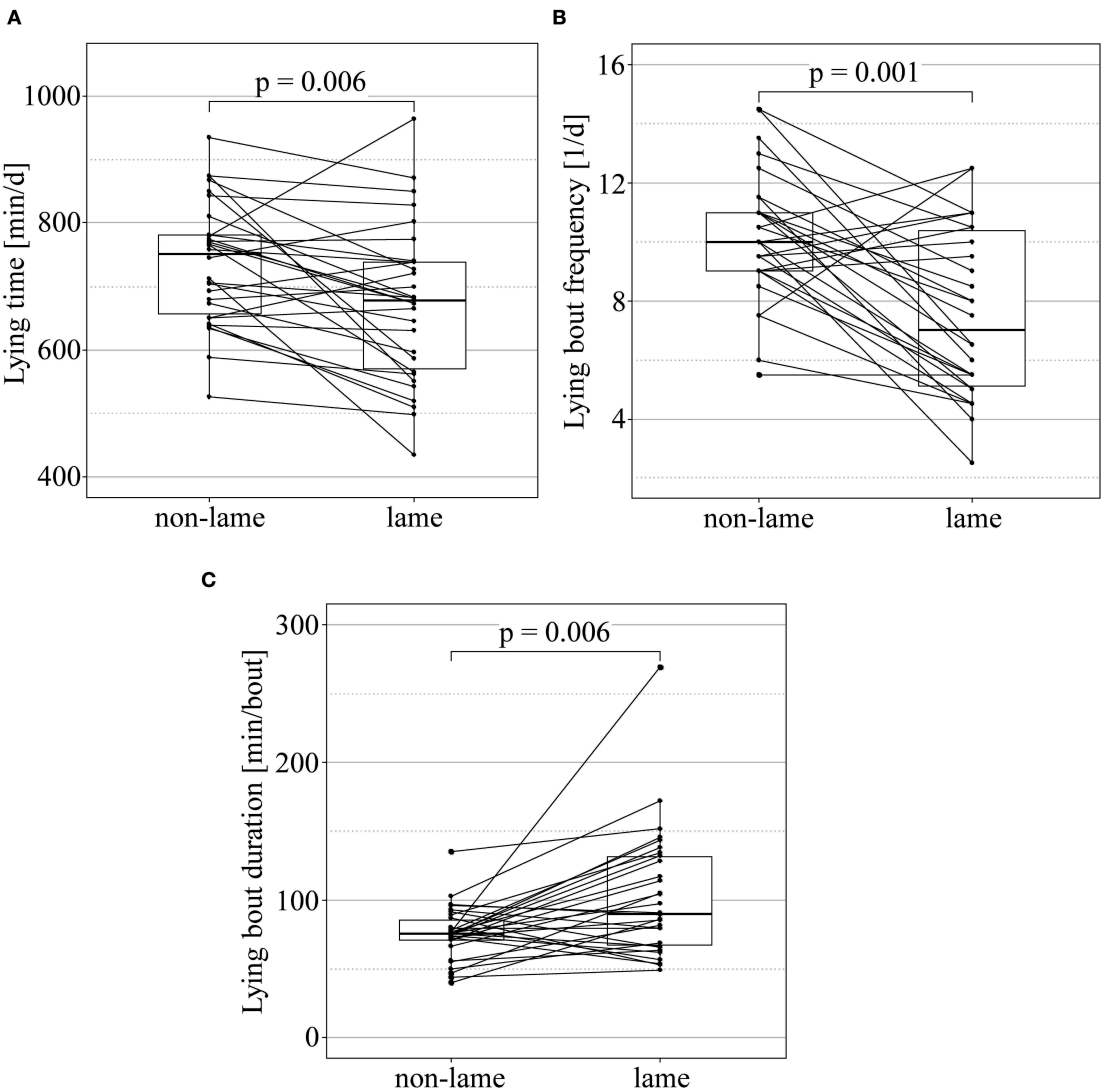
Lying behavior of non-lame (final locomotion score < 2.25) and lame cows [final locomotion score ≥ 2.25, according to Flower and Weary (39)]. Paired boxplots of lying time **(A)**, lying bout frequency **(B)**, and lying bout duration **(C)** of non-lame and lame cows, and associations between lameness and behavioral outcome tested in paired t-tests. *p*-values adjusted according to Benjamini and Hochberg (51). Each line between non-lame and lame represents a matched case-control pair.

**Figure 3:**
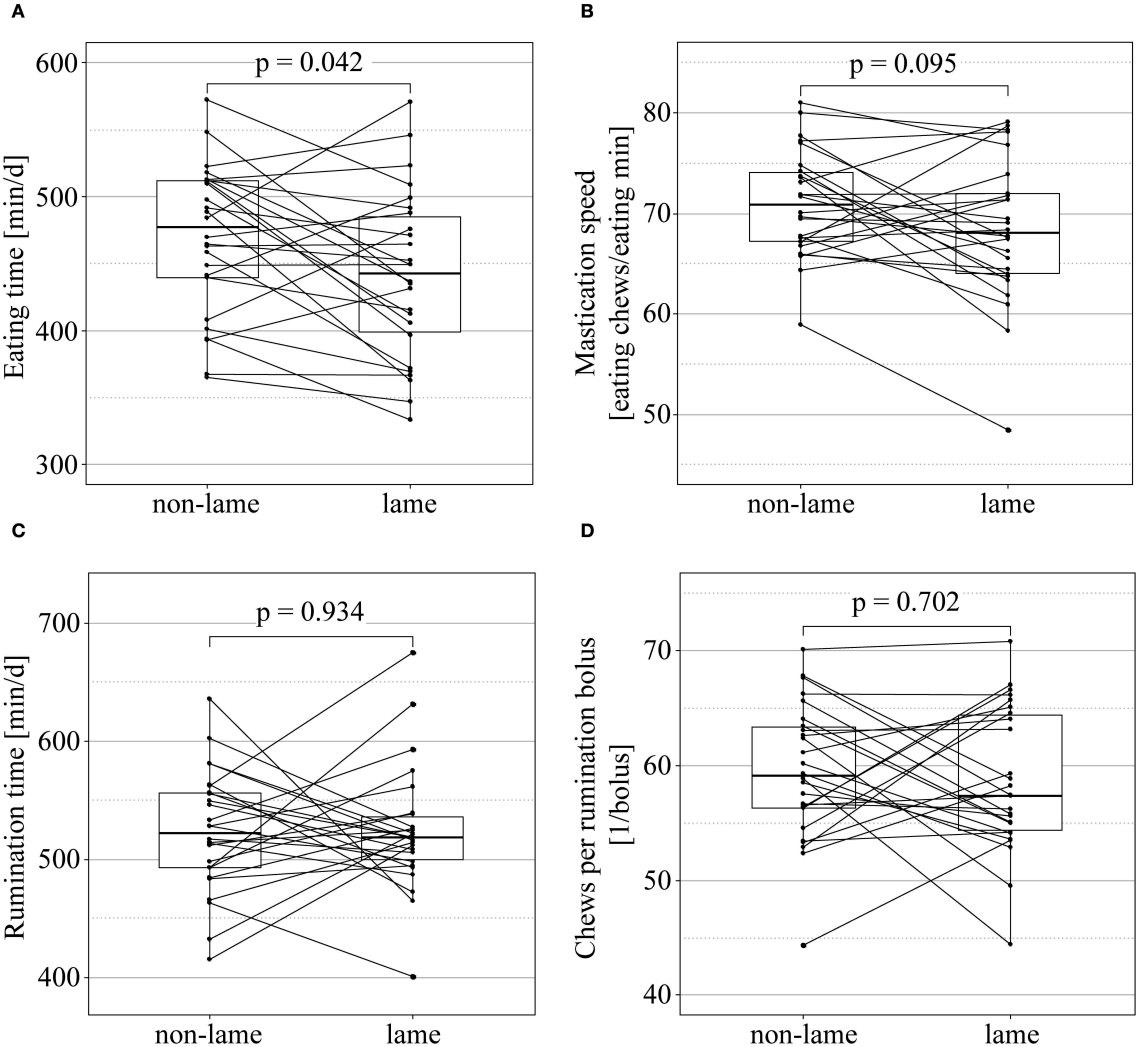
Eating and rumination behavior of non-lame (final locomotion score < 2.25) and lame cows [final locomotion score ≥ 2.25, according to Flower and Weary (39)]. Paired boxplots of eating time **(A)**, mastication speed **(B)**, rumination time **(C)**, and number of chews per rumination bolus **(D)** of non-lame and lame cows, and associations between lameness and behavioral outcome tested in paired t-tests. *p*-values adjusted according to Benjamini and Hochberg (51). Each line between non-lame and lame represents a matched case-control pair.

The lying and the eating and rumination behavior of non-lame controls and lame cases is visualized in Figures 2 and 3, respectively. The lying time was significantly shorter in lame cows compared to their non-lame controls (mean_diff_ = −64.7 min, CI_95%_ [−104.4–−24.9] min; paired *t*-test: df = 29, *t* = −3.3, *p* = 0.006). The lying bout frequency was significantly lower in lame cows compared to their non-lame controls (mean_diff_ = −2.6 bouts, CI_95%_ [−3.8–−1.4] bouts; paired *t*-test: df = 29, *t* = −4.4, *p* = 0.001), and the mean lying bout duration significantly longer (mean_diff_ = 26.7 min per bout, CI_95%_ [10.1–43.4] min per bout; paired *t*-test: df = 29, *t* = 3.3, *p* = 0.006). The eating time was the only halter-derived variable that was significantly different between cases and controls; lame cows had a significantly shorter eating time compared to their non-lame controls (mean_diff_ = −27.7 min, CI_95%_ [−51.5–−4.0] min; paired *t*-test: df = 25, *t* = −2.4, *p* = 0.042).

**Figure 4:**
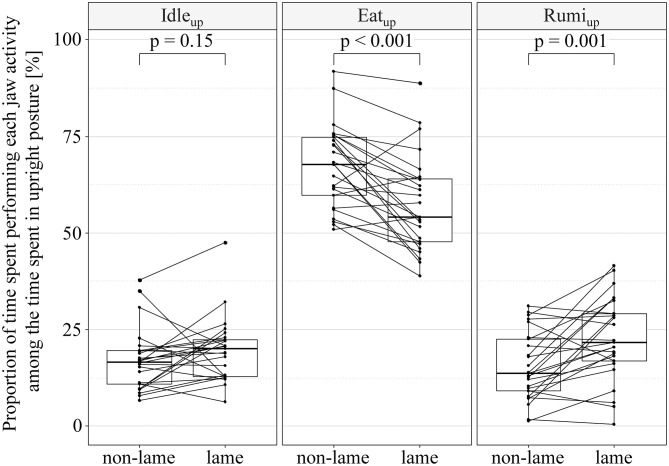
Jaw activities of non-lame (final locomotion score < 2.25) and lame cows [final locomotion score ≥ 2.25, according to Flower and Weary (39)] performed in upright posture. Paired boxplots of proportions of the upright time spent idling (Idle_up_), eating (Eat_up_), or ruminating (Rumi_up_), and associations of lameness and Idle_up_, Eat_up_, and Rumi_up_ tested in *post-hoc* paired *t*-tests subsequent to a repeated measures analysis of variance. Each line between non-lame and lame represents a matched case-control pair.

**Figure 5:**
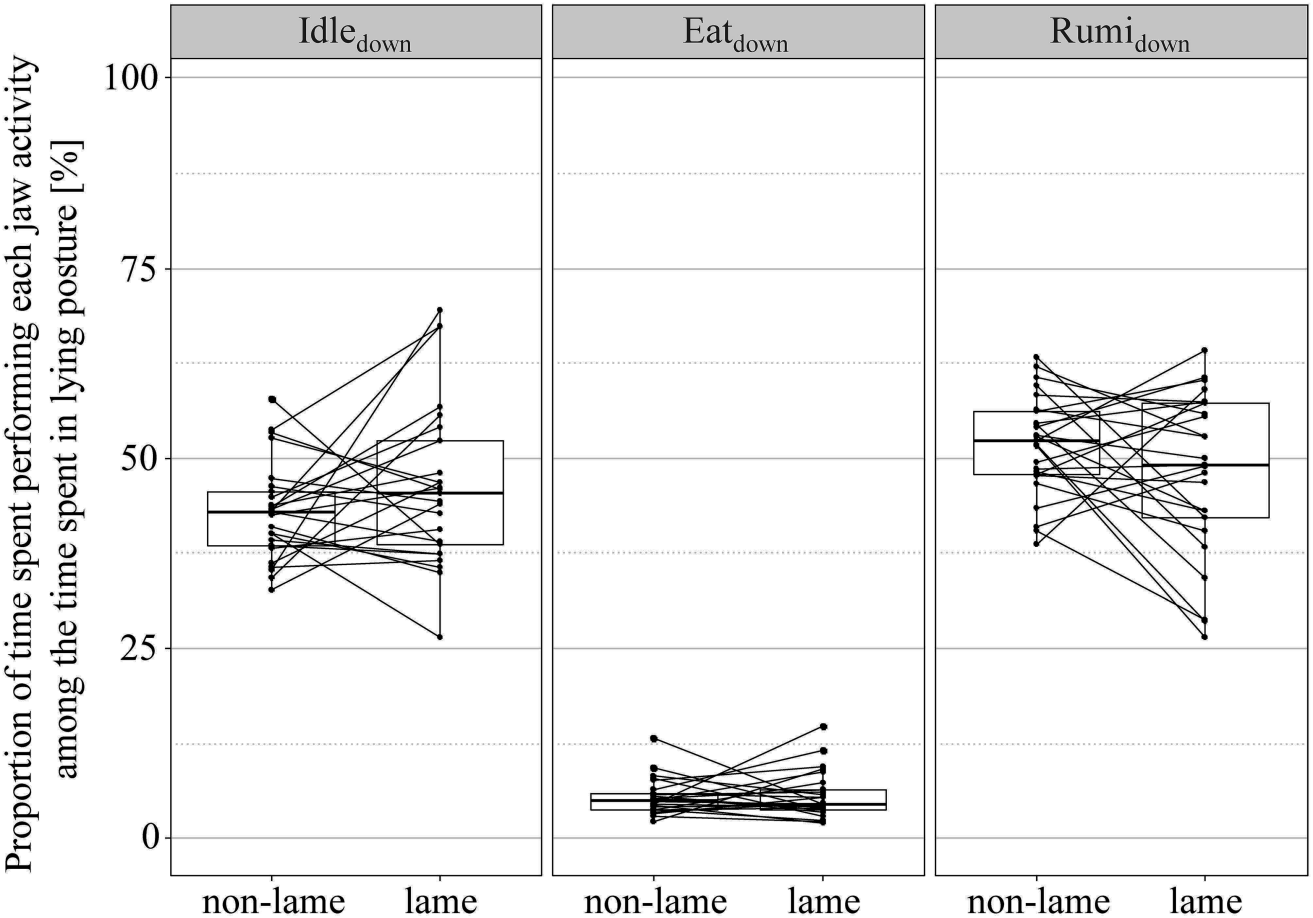
Jaw activities of non-lame (final locomotion score < 2.25) and lame cows [final locomotion score ≥ 2.25, according to Flower and Weary (39)] performed in lying posture. Paired boxplots of proportions of the upright time spent idling (Idle_down_), eating (Eat_down_), or ruminating (Rumi_down_). Each line between non-lame and lame represents a matched case-control pair.

The proportions of time spent performing each jaw activity (idling, eating, ruminating) among the time spent in either upright or lying posture are visualized in Figures 4 and 5. We found no evidence of a consistent effect of lameness on all jaw activity types (idling, eating, ruminating) performed in upright posture. However, the interaction term of activity type and lameness indicated a complex association of Idle_up_, Eat_up_, Rumi_up_ and lameness (rep. meas. ANOVA: df = 24, *p*_activitytype_ < 0.001, *p*_lameness_ = 0.996, *p*_activitytype*lameness_ < 0.001). Eat_up_ was lower (mean_diff_ = −9.7%, CI_95%_ [−14.5–−5.5]%; paired *t*-test: df = 24, *t* = −4.7, *p* < 0.001), and Rumi_up_ higher (mean_diff_ = 7.2%, CI_95%_ [3.2–11.1]%; paired *t*-test: df = 24, *t* = 3.7, *p* = 0.001) in lame cows compared to their non-lame controls. There was no evidence of a difference of Idle_up_ between cases and controls (mean_diff_ = 2.5%, CI_95%_ [−6.0–1.0]%; paired *t*-test: df = 24, *t* = 1.5, *p* = 0.150). The proportions of time spent performing each jaw activity among the time spent in lying posture was associated with the type of jaw activity, while lameness and the interaction term of lameness and jaw activity type were not (rep. meas. ANOVA: df = 24, *p*_activitytype_ < 0.001, *p*_lameness_ = 0.993, *p*_activitytype*lameness_ = 0.092).

## Discussion

Lame cows in tie stalls had fewer, longer lying bouts compared to their non-lame controls, but a shorter daily lying time. Further, lame cows had a shorter daily eating time compared to their non-lame controls and spent a larger proportion of their upright time ruminating.

### Lying Behavior

Lame cows had significantly fewer, but longer lying bouts compared to their non-lame controls. This is supporting our primary hypothesis and suggesting that lame cows might try to avoid the potentially painful procedure of rising and lying down. Haley et al. (14) reported that rising and lying down is generally aggravated for cows housed in tie stalls, since they experience space restrictions. Although these findings were not explicitly targeting lame cows, farmers report that they observe lame cows having more trouble with rising and lying down in their stalls compared to non-lame cows. Additionally, Chapinal et al. (53) stated, pain associated with a lameness-causing pathology “may reduce the willingness of a cow to stand up once she is lying down.” Sepulveda-Varas et al. (33) support these findings; however, other research groups found no clear association of lameness and lying bout frequency in free (23, 24, 29) or tie-stall-housed cows (11). By analyzing the data as matched case-control pairs and equalizing the preconditions that might influence behavior within pairs, significant differences between lame and non-lame cows could be detected in the current study.

Despite the longer duration of each lying bout, the lying time was significantly shorter in lame cows compared to their non-lame controls. On average, lame cows were lying about 1 h less, thus, standing 1 h more per day, compared to non-lame cows of the same farm, breed-type, and parity-group, which stands in contrast to our primary hypothesis that lame cows might rest longer to disburden the painful limb (5). In the current study, the stall surface was either a solid rubber mat or concrete, mostly covered with only little bedding, so that a good cushioning is questionable. Stall management and housing features had little influence on our results due to the matched case-control design; nevertheless, they must be considered when evaluating the situation of lame tied cows in practice. Since this study has a cross-sectional design, it cannot be determined, if the longer standing time is the cause or the effect of the lameness. The blood diffusion through the dermis of the claw is associated with lying and standing behavior of cows. “During inactive standing, there will be poor oxygenation and toxin removal” (54), thus longer standing times might increase the risk for claw lesions and lameness. However, the results of the current study suggest that lying down and to relieve a painful limb might not be the highest priority for lame tied cows but reducing the number of posture changes from lying to standing and vice versa seems to be important to reduce discomfort. The pressure to the legs and joints peaks during the process of lying down and rising, which might be particularly painful when the stall surface is poorly cushioned (55) or when the cow is lame.

Lying down and resting is often considered a measure of well-being and cow comfort (14, 56, 57); cows that experience some discomfort will probably avoid lying down. Consequently, the current study results suggest that slight and moderate lameness are likely to be associated with an impaired well-being in tied dairy cows.

### Eating and Rumination Behavior

The eating time in lame cows was shorter compared to their non-lame controls. In free stalls, lame cows tend to have a shorter eating time (5, 24, 58), because they avoid walking to (59) and standing at (5) the feed bunk to relieve their locomotor system. The lame cows avoid agonistic behavior toward other cows of the herd to assert a certain feeding place, especially in overstocked free stalls (60), and therefore spend as little time at the feed bunk as possible. However, these factors seem to be of minor importance in tie stalls, since tie-stall-housed dairy cows have their individual feeding place right in front of their individual stall, do not need to cover long distances to the feed bunk or to defend their feeding place toward higher-ranked cows. Furthermore, the assumption that lame cows might prefer lying over standing, secondarily resulting in a reduced eating time cannot be supported by the data of the current study, since the lame cows had a longer daily standing time but still a shorter eating time compared to their non-lame controls. Accordingly, it seems likely that the reduced eating time in the lame cows of the current study is a direct result of the painful process causing the lameness.

We found no difference in the mastication speed between lame and non-lame cows. A faster mastication speed was described as a potential compensation mechanism for the reduced eating time in lame free-stall-housed cows (5, 24, 58), as a faster mastication speed is expected to be associated with a higher feed uptake. The specific feed uptake of each cow was not quantified in the current study; however, the lower eating time and the unaltered mastication speed are expected to result in decreased amounts of feed taken up by lame cows.

Although the mean daily decrease of half an hour of eating time per day in lame cows appears to be relatively low, it has to be considered that especially slight lameness is often recognized and treated with a delay (26, 61); thus, in the long term, the effect of reduced eating time adds up over time. In the current study, lame cows had a statistically lower body condition score (41) compared to their non-lame controls, although the mean_diff_ of −0.13 points should at most be interpreted as a tendency. However, a low body condition score predisposes cows to become lame (62–64) and the reduced eating time and unaltered mastication speed in lame cows might lower the chances for thin cows to regain an optimal body condition.

The rumination time and the number of chews per rumination bolus were similar between lame cows and their non-lame controls in the current study. Beer et al. (24) reported rumination activities to be lower in lame compared to non-lame free-stall-housed dairy cows; a finding that we could not confirm in tie-stall-housed cows. The impact of lameness seems to be subordinated to the effect to the physical structure of the feed, which mainly influences the rumination activity in tied cows (32) and was similar within matched pairs.

### Jaw Activities in Either Upright or Lying Posture

Lame cases spent a significantly larger proportion of their upright time ruminating compared to their non-lame controls, while there was no evidence of an association of slight and moderate lameness and the proportions of time spent performing each jaw activity among the time spent in lying posture.

Eating is a high-priority behavior of dairy cows (65) which is predominantly performed in upright posture. In the current study, the lower Eat_up_ in lame cows is a logical consequence of the shorter absolute eating time and longer absolute standing time of lame compared to non-lame cows. However, the decreased Eat_up_ was not compensated for by Idle_up_, but rather by Rumi_up_. Haley et al. (14) described extended times of standing idle as a sign of poor cow comfort and well-being, especially in stalls with poor cubicle design and uncomfortable lying areas. The current results render no evidence that slight and moderate lameness are also associated with increased times of standing idle. Interestingly, Rumi_up_ was significantly higher in lame cows compared to their non-lame controls, although the absolute rumination time was similar between cases and controls. Rumination is often assumed to be associated with good animal health (66) and well-being, especially when the cows are ruminating while lying (4); by implication, ruminating in an upright position is assumed to be associated with poor cow comfort and well-being. Further, an increased time of ruminating upright was recently reported in cows with an impaired well-being due to a prolonged milking interval, which was interpreted as a potential replacement activity for a reduced eating time (67). The fact that the lame cows in the current study spent more time Rumi_up_ instead of Rumi_down_ might be another measure of the impact of lameness on the well-being of tied dairy cows.

### Strengths and Limitations of the Current Study

The individual variation in behavior expression was described earlier for tied dairy cows, aggravating comparisons on the cow level (11). Consequently, we matched case-control pairs on the criteria farm, breed-category, and parity-group, as all of them influence the behavior of dairy cows (32, 36, 68), aiming to reduce the uncontrolled individual variation in behavior expression. Obtaining a large enough sample size of matched pairs was a challenge in Swiss tie stalls due to the typically small herd size of 22 cows per Swiss dairy herd (69) and the low Swiss lameness prevalence of 14.8% (70). Since the lactation stage is reported to influence behavior (34, 35), the additional matching criterion days post-partum would have been desirable but only possible in larger herds. In our study, the lame cases were tendentially in an earlier lactation stage compared to the non-lame controls; however, we suspected the effect of the mean_diff_ of −44 days on the behavior to be rather small.

Despite the statistically significant differences in lying time, lying bout frequency and duration, eating time and upright activities between cases and controls, the association between lameness and behavior could not be confirmed among all enrolled pairs. An individual, within-species variation in pain tolerance was supposed before (71); this might cause individually different effects of lameness on behavior and well-being and is supported by the results of the current study. Longitudinal studies that follow up the behavior of individual cows would allow for analyzing individual baseline measures of each behavior and the relation of behavioral changes over time. Although, such studies are highly elaborative, they would enable intraindividual comparison, account for an individual pain tolerance and behavior expression and create a deeper understanding of cow behavior.

Most Swiss tied dairy cows are brought to Alpine pastures in summer and need to cover long distances walking, so that most farmers are aware of the importance of a healthy locomotor system in their cows. In our sample, this awareness might be overrepresented due to the voluntary participation of farmers, resulting in a low prevalence and rather mild severities of lameness. Regardless of these preconditions, we primarily aimed to mainly enroll slightly and excluded severely lame cows as cases. It is known that slightly lame cows who are unfortunately often detected and treated with a delay (26, 61) would have the best prognosis on healing after a correct and timely treatment (28, 72). Compared to previous studies (73, 74), the cut-off of a final LS ≥ 2.25 to qualify a cow as a lame case appears to be a very stringent admission of the locomotion scoring according to Flower and Weary (39). It seems obvious that severely lame cows (LS ≥ 4) behave differently compared to non-lame cows and experience discomfort, regardless of the apparent housing system. The current study highlights that even slight lameness is associated with alterations of the behavior and probably an impaired well-being of tied dairy cows. This underlines the need to target early onsets and mild forms of lameness in programs that aim to improve lameness detection and reduce lameness severity and prevalence.

## Conclusions

Slight and moderate lameness are associated with alterations of the eating, rumination, and lying behavior of lame dairy cows housed in tie stalls. Our study highlights that even in stall systems with comparably low demands to the cows' locomotor systems, lameness is associated with alterations of behavior. Slight and moderate lameness are likely to be associated with an impaired well-being to an amount that can be objectively measured and quantified in an interindividual comparison between matched pairs of non-lame controls and lame cases. To reduce the impact of lameness on the behavior and well-being of dairy cows housed in tie stalls, intensified efforts should be made to timely detect and adequately treat lame cows in order to continuously reduce the lameness prevalence in tied dairy herds.

## Data Availability Statement

The original contributions generated for the study are included in the article/Supplementary Material, further inquiries can be directed to the corresponding author/s.

## Ethics Statement

The animal study was reviewed and approved by Veterinary Office of the Canton Bern, Switzerland (approval number: 29518). Written informed consent was obtained from the owners for the participation of their animals in this study.

## Author Contributions

The conceptualization and administration of the study were performed by JB and AS. The data curation was performed by JB, BV, RA, and RR. Formal analysis was performed by JB, BV, and DS. The investigation was performed by JB, RA, and RR. The methodology was planned by JB, BV, DS, KM, and AS. AS was supervising the project. JB visualized the data and wrote the first draft of the manuscript. JB, BV, DS, KM, and AS reviewed and edited the manuscript to be submitted. All authors approved the submitted version.

## Conflict of Interest

The authors declare that the research was conducted in the absence of any commercial or financial relationships that could be construed as a potential conflict of interest.
