# Supplementary material for: Slightly and Moderately Lame Cows in Tie Stalls Behave Differently From Non-lame Controls. A Matched Case-Control Study
Source: Front Vet Sci. 2020 Dec 17;7:594825. doi: 10.3389/fvets.2020.594825 (PMC7773726; doi:10.3389/fvets.2020.594825)
Supplement: Supplementary file 1 [file Table_1.pdf]

Supplementary Table 1. Definitions of assessed behavioral parameters. Parameters derived from RumiWatch® halters and pedometers (RumiWatch®, ITIN+HOCH GmbH, Fütterungstechnik, Liestal, Switzerland), adapted from Beer et al. (2016).

**Eating and rumination behavior**	**Definition**
Idle time[1]	Idle time in min per day; time spent for jaw movements classified as neither eating nor ruminating
Eating time[a,1]	Eating time in min per day
Eating chews[a,1]	Number of eating chews per day
Mastication speed[c,1]	Number of eating chews per eating min, "Eating chews" divided by "Eating time"
Rumination time[a,1]	Rumination time in min per day
Chews per rumination bolus[a,1]	Mean number of rumination chews per rumination bolus
**Lying behavior**	
Lying time[b,1]	Lying time in min per day
Lying bout frequency[b,1]	Number of lying periods > 50 s per day in counts per day
Lying bout duration[c,1]	Lying bout duration in min per bout, "Lying time" divided by "Lying bout frequency"
Total upright time[2]	Sum of upright time (standing time + walking time) in min per day, including all 5 min intervals without posture change from lying to standing or vice versa
Total lying time[2]	Sum of lying time in min per day, including all 5 min intervals without posture change from lying to standing or vice versa
**Jaw activities performed in either upright and or posture**	
$Idle_{up}$[2]	Proportion of time spent idling among the time spent in upright posture, in %
Eat_{up}[2]	Proportion of time spent eating among the time spent in upright posture, in %
$Rumi_{up}$[2]	Proportion of time spent ruminating among the time spent in upright posture, in %
$Idle_{down}$[2]	Proportion of time spent idling among the time spent in lying posture, in %
Eat_{down}[2]	Proportion of time spent eating among the time spent in lying posture, in %
$Rumi_{down}$[2]	Proportion of time spent ruminating among the time spent in lying posture, in %

[a]variable validated by Zehner et al. (2017)
[b]variable validated by Alsaaod et al. (2015)
[c]proportion of validated variables
[1]derived from 24 h summaries of the RumiWatch converter V0.7.3.6
[2]derived from 5 min summaries of the RumiWatch converter V0.7.3.6
