# Supplementary material for: Slightly and Moderately Lame Cows in Tie Stalls Behave Differently From Non-lame Controls. A Matched Case-Control Study
Source: Front Vet Sci. 2020 Dec 17;7:594825. doi: 10.3389/fvets.2020.594825 (PMC7773726; doi:10.3389/fvets.2020.594825)
Supplement: Supplementary file 2 [file Data_Sheet_1.PDF]

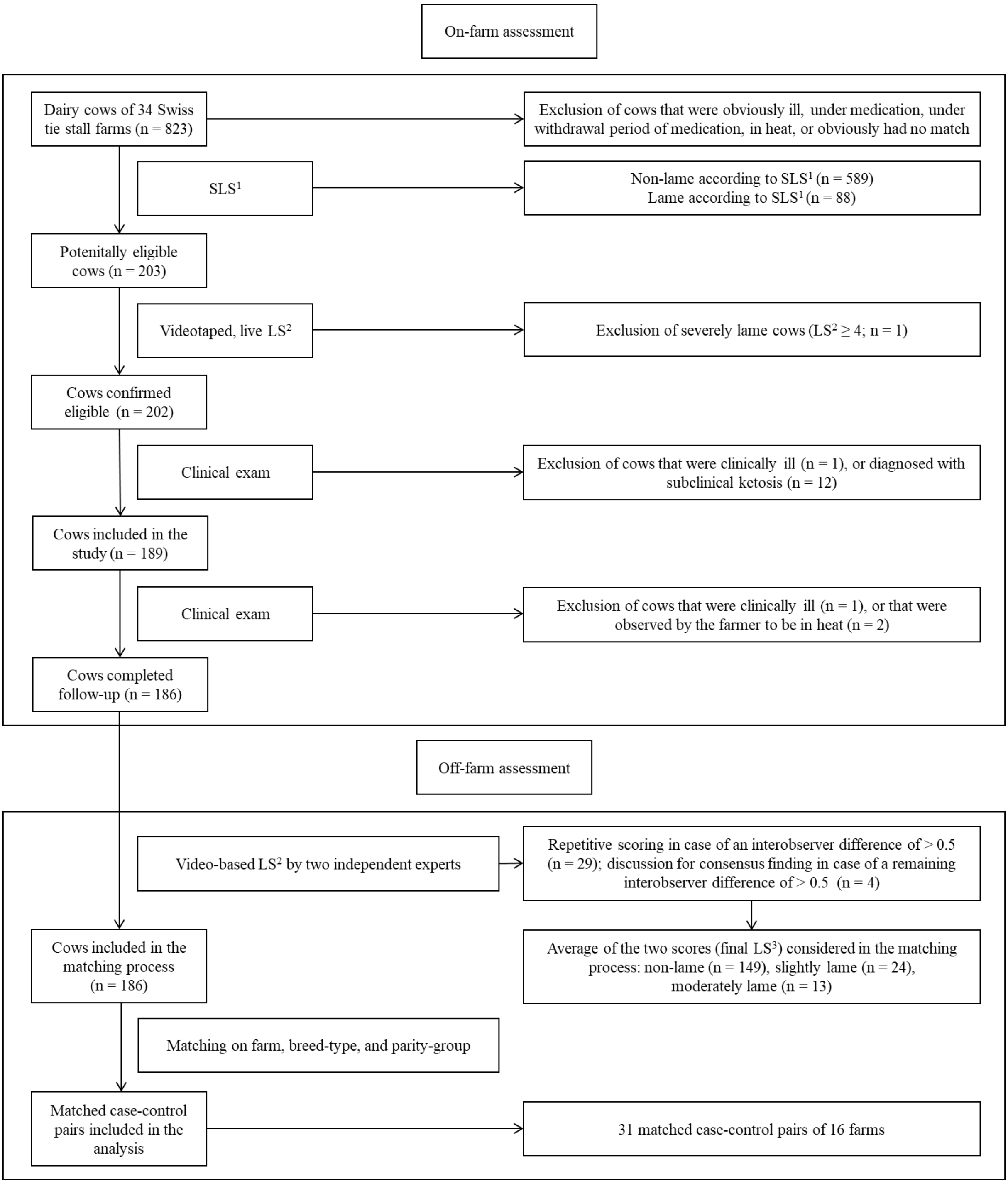

Supplementary Figure 1. Cow-selection procedure. Flowchart of the on and off-farm procedures to build matched case-control pairs, each formed by one lame and one non-lame cow of the same farm, breed-category, and parity-group. [1]SLS: stall lameness score according to Leach et al. (2009). [2]LS: locomotion scoring according to Flower and Weary (2006). [3]final LS: averaged LS (Flower and Weary, 2006) of the two independent observers; non-lame: final LS < 2.25; slightly lame: final LS 2.25 – 2.75; moderately lame: final LS ≥3.0.
